# Changes in participatory and societal outcomes during the waiting period for cochlear implantation – an observational study

**DOI:** 10.1007/s00405-024-08981-7

**Published:** 2024-09-26

**Authors:** Hugo G.B. Nijmeijer, N. Philpott, GJ van der Wilt, A. R.T Donders, E. George, R. Boerboom, J. H.M. Frijns, M. Kaandorp, W. J. Huinck, E. A.M. Mylanus

**Affiliations:** 1 Department of Otorhinolaryngology, Radboud university medical center, Nijmegen, The Netherlands; 2 Donders Institute for Brain, Cognition and Behaviour, Radboud University, Nijmegen, The Netherlands; 3 Department for Health Evidence, Radboud university medical center, Nijmegen, The Netherlands; 4 Department of ENT/Audiology, School for Mental Health and Neuroscience, Maastricht University Medical Centre, Maastricht, The Netherlands; 5 Department of Otorhinolaryngology, Head and Neck Surgery, University Medical Center Utrecht, Utrecht University, Utrecht, The Netherlands; 6 Department of Otorhinolaryngology, Leiden University Medical Center, Leiden, The Netherlands; 7 Leiden Institute for Brain and Cognition, Leiden, The Netherlands; 8 Department of Bioelectronics, Delft University of Technology, Delft, The Netherlands; 9 Department of Otolaryngology - Head and Neck Surgery, section Ear & Hearing, Amsterdam University Medical Center location Vrije Universiteit, Amsterdam Public Health research institute, Amsterdam, The Netherlands

**Keywords:** Cochlear implantation, Hearing loss, Quality of life, Outcomes, Waiting time, Employment

## Abstract

**Introduction:**

Various factors, including an aging population and expanding eligibility criteria, may increase the demand for cochlear implants (CIs), potentially resulting in longer waiting times. In most Dutch CI centers, the time between referral and surgery exceeds 6 months. Clinical experience suggests that during the waiting period for cochlear implantation, hearing and communication difficulties increase. Simultaneously, there is an interest in outcomes more closely aligned with patient values and needs, which resulted in the SMILE (Societal Merit of Interventions on hearing Loss Evaluation) study. This paper presents results on observed changes in societal and participatory outcomes during waiting time in participants with a time to CI surgery exceeding 6 months.

**Methods:**

SMILE is a prospective multi-center study including 232 individuals who were referred for unilateral CI. Continuous and nominal data from multiple questionnaires, sent immediately after referral and shortly before surgery, were analyzed by computing differences, Cohen’s D, and odds ratios.

**Results:**

Of the total 232 participants, 102 had a time between inclusion and surgery exceeding 6 months. Of these, 89 had (partially) filled out surveys at both time points. Of all the domain scores 55% did not show differences between timepoints. All Cohen’s D estimates were relatively small, ranging from − 0.298 to 0.388 for those outcomes that showed noteworthy changes.

**Conclusion:**

Waiting time from referral to surgery, even though exceeding 6 months, was observed to not seriously affect non-clinically-prioritized patients in an adverse way. Future investigations should identify subgroups on tolerable waiting times regarding short- and long-term outcomes.

**Trial registration:**

Trial registration number at ClinicalTrials.gov: NCT05525221, 25-8-2022.

**Supplementary Information:**

The online version contains supplementary material available at 10.1007/s00405-024-08981-7.

## Introduction

Cochlear implantation (CI) is considered a cost-effective intervention in the rehabilitation of severe to profound hearing loss [1, 2]. However, the utilization rate for cochlear implants is relatively low, indicating that only a limited percentage of individuals who might benefit from CI eventually get implanted [3–6]. There are multiple factors affecting CI utilization and accessibility of CI healthcare services [7–9]. As a result of increasing clinical experiences and ongoing technological advances, the CI eligibility criteria are continuously reevaluated, leading to an increase in CI demand.

Simultaneously, the healthcare system is changing with increasing pressure and limited resources [10], which amongst other factors could result in increasing waiting times. Depending on the health issue, long waiting times may negatively affect outcomes and even reduce postoperative health gains compared to timely interventions [11, 12]. In Dutch CI centers, individuals with severe to profound hearing loss usually experience a time span from CI referral to surgery that exceeds 6 months, although this period may fluctuate. Clinical experiences in CI care suggest that the waiting time for hearing rehabilitation might have negative consequences for individuals with severe to profound hearing loss. During the waiting period the already profound hearing difficulties often further deteriorate, which may result in further social isolation. This relates to the conceptual 3-stage model of auditory performance introduced by Blamey et al. on how auditory performance is affected by the duration of hearing loss, auditory deprivation and learning plasticity [13]. To date, there is limited evidence regarding the consequences of waiting on broader participatory and socioeconomic outcomes in adults with severe to profound hearing loss. In addition, there is a growing interest in value-based healthcare and the desire to gain insights into outcomes that are more closely aligned with individuals’ values and needs [14, 15].

This paper presents the first results of the Societal Merit of Intervention on Hearing Loss Evaluation (SMILE) study [16]. The primary aim of this paper is to investigate short-term changes in participatory and societal outcomes during the waiting time for CI in individuals with a time to CI surgery exceeding 6 months.

## Methods

The SMILE study is a multi-center prospective interventional cohort study in the Netherlands. In this study, individuals with hearing loss who were willing to participate were included immediately after referral to the ENT Departments and/or Audiological Centers for consideration of cochlear implantation. Measurements are taken at several time points: at study inclusion (baseline), right before CI surgery, and 1, 2, and 3 years after CI surgery. For a complete description of the SMILE study and its methodology, we refer to the SMILE protocol [16]. The primary aim of this paper is to investigate short-term changes in participatory and societal outcomes during the waiting time for CI in individuals with more than 6 months of waiting time. In addition to changes related to (un)employment, a secondary aim is to compare the unemployment status of SMILE participants at baseline to the general Dutch population.

### Study design and setting

Study participants described in this paper were recruited in the participating university medical centers (UMCs) of Maastricht, Amsterdam, Utrecht, Leiden and Nijmegen between August 2020 and December 2022. Castor EDC (Electronic Data Capture) was used for capturing data and administrating validated questionnaires by using Castor’s online survey tool [17]. Castor enables reliable and constant data capturing, which is stored for at least 15 years.

To explore changes during the time until surgery, data was gathered at two preoperative time points: The baseline measurement (T0), conducted immediately after referral and signing informed consent and the second measurement (T1), scheduled two weeks before CI surgery. This second measurement was only collected if the time between study inclusion and surgery was longer than 6 months.

## Participants

### Inclusion criteria

The following in- and exclusion criteria for study participation were maintained.

1. Age 18 years or older.
2. Bilateral severe to profound postlingual sensorineural hearing loss (as defined by WHO criteria > 61 dB loss), *or a ski-slope threshold with severe to profound sensorineural hearing loss in the high frequencies, and referred to a university medical center for potential eligibility for CI.
3. Eligible for CI based on clinical criteria, specifically:
  - best aided phoneme score ≤ 70% (*in case of rapid progressive hearing loss ≤ 80%) at 65/70dB SPL.
  - communication needs expressed by the individual during the CI intake procedure.
4. Native speakers of the Dutch Language.

### Exclusion criteria

The following exclusion criteria were maintained for the study.

1. Presence of an underlying syndrome or psychiatric disorder.
2. Incapable of performing (un)paid labor, due to non-hearing related factors.
3. Pre-lingual profound hearing loss or deafness.
4. Children (0–18 years).
5. Any condition that may hamper a complete insertion of the electrode array or a normal rehabilitation with the cochlear implant (severe otosclerosis or neurologic deficits).

To make study criteria in line with current Dutch clinical CI eligibility criteria, the in-and exclusion criteria presented in the study protocol were slightly adapted (indicated by *).

Participants were divided into two groups: Group 1 comprised the (potential) working population, consisting of adults aged between 18 and 65 years. Group 2 comprised adults over the age of 65 years, approximately representing the retired population.

Given the observational nature of the study, sample size was not determined for this specific SMILE research question. Instead, we aimed to include and measure as many individuals as possible to obtain precise estimates of changes during the waiting time.

Variables.

Relevant outcomes, the measurement tools utilized, and their interpretations are presented in Table 1. Data gathered in the clinical CI process were used to describe participant characteristics and to identify potential outliers.

**Table 1:** Overview of the questionnaires and their outcome domains

Aim	Tool	Domains	Interpretation of scores
Participation and autonomy	IPA [18]	- Autonomy indoors - Autonomy outdoors - Family role - Social life and relationships - Work and education	Higher score indicates more difficulties or problems in participation and autonomy.
Communication strategies and personal adjustments	Dutch CPHI [19]	Use of communication strategies - Maladaptive behaviors - Verbal strategies - Non-verbal strategies Personal adjustment to hearing impairment: - Self-acceptance - Acceptance of loss - Stress and withdrawal	Scores reflect behaviors related to hearing impairment, with lower scores indicating a higher potential for coping difficulties compared to higher scores.
Health Related Quality of Life	NCIQ [20]	The physical domain: - Basic sound perception - Advanced sound perception - Speech production Psychological domain: - Self-esteem Social domain - Activity limitations - Social interactions	Higher scores indicate better results.
	Third party Hearing loss related quality of life (HII-SOP) [21]	20 items which are divided over three subscales: - Communication strategies - Relationships and emotions - Social impact	Lower scores indicate less third-party disability. Interpretation of scores for third party disability: < 20 = ‘No’ 20–39 = ‘Mild’ 40–59 = ‘Moderate’ > 60 = ‘Severe’
	HUI3 [22, 23]	Total score and 8 attributes: Vision, hearing, speech, ambulation, dexterity, emotion, cognition and pain.	Quality of Life scoring system that provides utility (preference) scores. Dead = 0 and perfect health = 1. Higher scores indicate greater health related quality of life.
	EQ-5D [24, 25]	Total score and five dimensions: mobility, self-care, usual activities, pain/discomfort and anxiety/depression.	Quality of Life scoring system. Values are anchored at 1 (full health) and 0 (a state as bad as being dead).
Capability	ICECAP-A [26–28]	Total score and five attributes; - Attachment - Stability - Achievement - Enjoyment - Autonomy	A score of 0 indicates no capability. A score of 1 indicates full capability.
Work: - Capability - Experience	LWC [29, 30]	Values included in the questionnaire. 1) Use of knowledge and experience 2) Development of knowledge and experience 3) Involvement in important decisions 4) Creating meaningful social contacts at work 5) Setting personal goals 6) Good income 7) Contributing to something valuable Each item is asked and scored in 3 sub-items. A) How important is this value in your work? B) Does your work situation enables you to realize this value? C) Do you actually realize this value?	A value is part of the capability set (score of 1) if: the value is important (A = 4 or 5), the value can be realized (B = 4 or 5) and the value is actually realized (C = 4 or 5). In all other combination the value is not part of the capability set (score of 0). The sum of score shows the total capability set from a minimum of 0 to a maximum of 7. Higher scores indicate higher sustainable employment.
	QEEW [31]	Three scales - Participation/say - Relationship with co-workers - Need for recovery	Higher scores indicate a worse state on the given domain.

IPA: Impact on Participation and Autonomy questionnaire, CPHI: Communication Profile for the Hearing Impaired, NCIQ: Nijmegen Cochlear Implant Questionnaire, HII-SOP: Hearing Impairment Impact-Significant Other Profile, HUI3: Health Utilities Index Mark 3, EQ-5D: generic quality of life instrument developed by the EuroQoL Group, ICECAP-A: ICEpop (Investigating choice experiments for the preferences of older people) CAPability measure for Adults, LWC: List Work Capabilities, QEEW: Questionnaire on the Experience and Evaluation of Work [18–31]

### Bias

An approach to reduce response bias involved the administration of online surveys instead of paper questionnaires. All participants completed the online surveys unless a paper version was requested (total of 13 requests, 12 at T0 and 1 at T1). In the online survey, the items that are relevant to everyone’s life were mandatory so that participants were compelled to provide the answer that best reflected their situation. This reduced the number of missing values. Using online survey tools also has the advantage that it reduces the risk of human error during data capturing, compared to paper questionnaires. Reminders were sent after approximately 2 weeks to encourage completion of the surveys. In case a participant did not complete the T1 survey before surgery, they had time to complete it until CI activation (approximately 3 weeks post-implantation) before the survey was locked. To ensure consistent data collection, the audiological protocols of each center were compared and adjusted if needed before the start of data collection.

### Statistical analysis

Descriptive statistical analyses were performed to summarize the baseline characteristics of participants, including age, sex, etiology of hearing loss, duration of hearing loss, living situation (household composition), and educational background.

To provide insights into the primary aim of this paper, we analyzed data of participants who completed the questionnaires to the extent that valid (sub-domain) scores could be computed at T0 and T1. To explore the differences in outcomes between T0 (inclusion) and T1 (short before surgery) we computed the mean of the differences and the measure of effect size, Cohen’s D, both with related 95% confidence intervals. For categorical variables, we computed odds ratios and corresponding 95% confidence intervals.

Furthermore, unemployment percentages at T0 in the SMILE population are calculated and compared to the general Dutch population by using employment data of the second quartile of 2023 of Statistics Netherlands [32]. To account for differences in the age distribution between the SMILE population and the general population, standardized unemployment ratios were calculated.

Additional analyses of retrospectively defined sub-groups are provided to assess the comparability at T0 regarding the outcomes of interest. These groups are: (1) implanted versus the not-implanted and (2) longer versus shorter than 6 months waiting for CI surgery.

All analyses were conducted by using R studio software. Presented p-values in the supplementary material tables are the results of continuity corrected Wilcoxon signed rank tests and Wilcoxon rank sum tests for the primary (paired) and additional (unpaired) analysis, respectively. P-values were not corrected for multiple testing, and given the observational nature of the study, they should be interpreted with caution.

## Results

Participants and descriptive data.

**Fig. 1:**
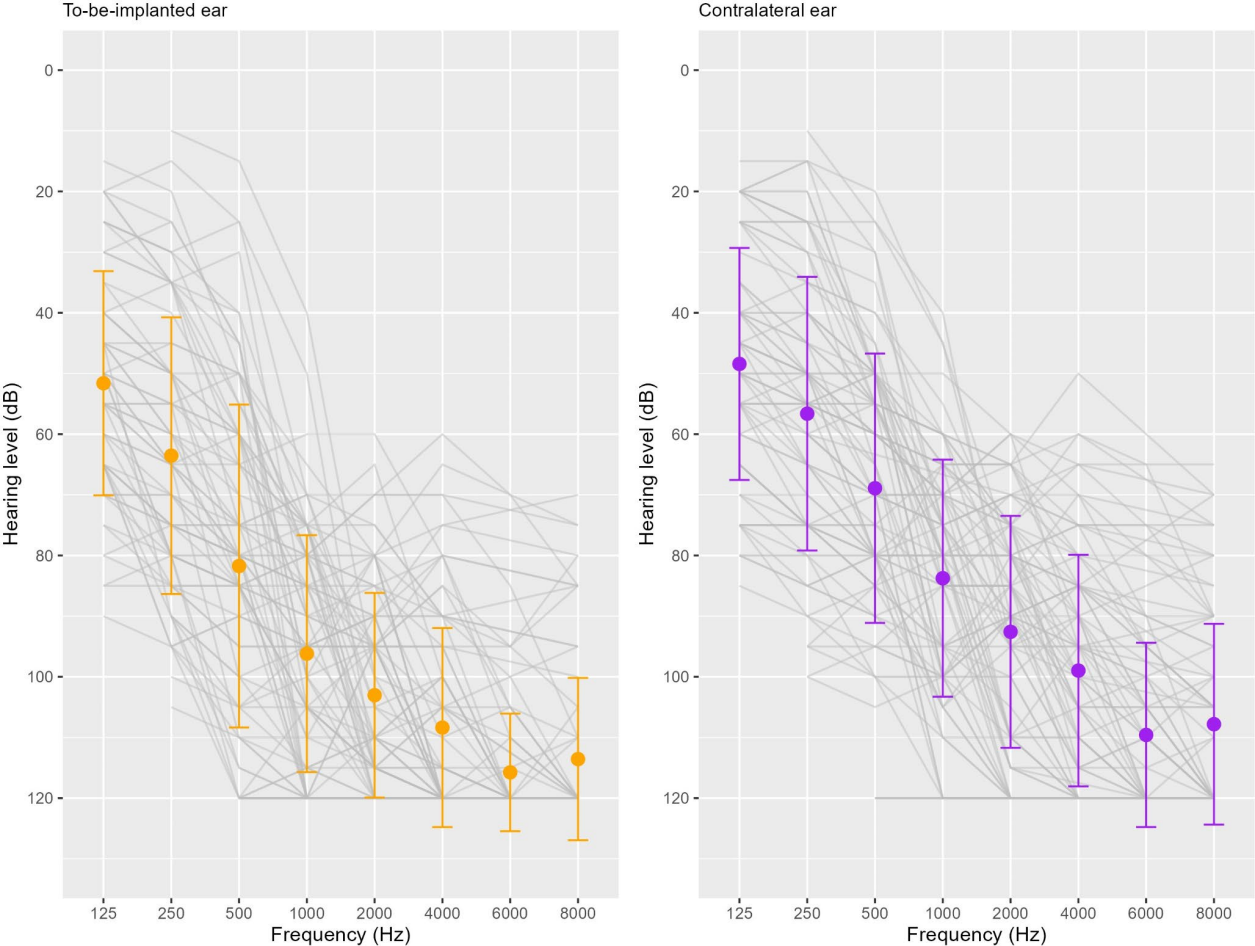
Baseline (T0) un-aided average audiograms (+/- 1 SD), grey lines present individual thresholds (*N* = 89). The thresholds only represent responses to stimuli up to a maximum intensity of 120 dB. Several participants did not respond at maximum intensity (120 dB) for at least one frequency. Stimuli presented between 500 and 8000 Hz that were not heard at 120 dB were set at 120 dB. Due to the risk of vibrotactile responses at the lower frequencies, stimuli at 125 and 250 Hz that were not heard are not depicted and not included in the calculation of the averages

Individuals were routinely asked to participate in the study during the process of referral for an intake appointment at the CI clinic. Approximately 451 participants were contacted in the participating centers, 178 did not respond and 41 did not consent to study participation, resulting in 232 included participants (148 from Radboudumc, 30 from MUMC, 6 from AUMC, 30 from UMCU and 18 from LUMC). Some of these included participants dropped out of the study (either preoperatively *n* = 7 or postoperatively *n* = 10), did not meet the study criteria (*n* = 16), or did not continue in the CI trajectory at the time of analysis (*n* = 59). There were various reasons for drop-out: Some participants were considered not (yet) eligible for CI and others decided not to continue with the CI trajectory for various personal reasons (amongst others: fear of surgery, not the right time, other medical issues). Other reasons for ending participation before or after receiving a CI were time availability or inclusion in other research trials. Eventually, 150 participants received a CI, of which 102 had a waiting time between inclusion and implantation of more than 6 months. Eighty-nine participants had a waiting time longer than 6 months and (partially) completed both T0 and T1 questionnaires. These participants were included in our analysis and their baseline characteristics are presented in Fig. 1; Table 2. A more detailed table is provided in the supplementary material (Table S1).

**Table 2:** Baseline characteristics of SMILE participants who experienced a waiting time for surgery longer than 6 months and (partially) completed T0 and T1 surveys

	Under 65 (*N* = 39)	Over 65 (*N* = 50)	Total at T0 (*N* = 89)
**Age at inclusion (years)**			
Mean (SD)	54.7 (8.97)	72.4 (4.75)	64.7 (11.2)
Median [Min, Max]	58.0 [31.0, 64.0]	72.0 [65.0, 85.0]	67.0 [31.0, 85.0]
**Sex**			
Male	17 (43.6%)	28 (56.0%)	45 (50.6%)
Female	22 (56.4%)	22 (44.0%)	44 (49.4%)
**Time between inclusion and surgery (months)**			
Mean (SD)	11.4 (5.44)	11.2 (3.07)	11.3 (4.25)
Median [Min, Max]	10.6 [6.40, 33.7]*	11.5 [6.10, 17.9]	11.0 [6.10, 33.7]*
**Age of noticing hearing loss (years)**			
Mean (SD)	28.1 (16.2)	46.2 (17.6)	38.8 (19.1)
Median [Min, Max]	30.0 [0, 54.0]	50.0 [0, 79.0]	41.0 [0, 79.0]
Missing	5 (12.8%)	1 (2.0%)	6 (6.7%)
**Duration of hearing loss (years)**			
Mean (SD)	26.4 (13.2)	26.2 (18.0)	26.3 (16.1)
Median [Min, Max]	22.5 [9.00, 61.0]	20.0 [2.00, 80.0]	21.0 [2.00, 80.0]
Missing	5 (12.8%)	1 (2.0%)	6 (6.7%)
**Category etiology of hearing loss**			
Acquired	6 (15.4%)	10 (20.0%)	16 (18.0%)
Not-syndromal	10 (25.6%)	9 (18.0%)	19 (21.3%)
Syndromal	4 (10.3%)	2 (4.0%)	6 (6.7%)
Unknown	19 (48.7%)	29 (58.0%)	48 (53.9%)
**Paid or unpaid Work**			
Yes	30 (76.9%)	26 (52.0%)	56 (62.9%)
No	9 (23.1%)	24 (48.0%)	33 (37.1%)
**Paid Work**			
Yes	24 (61.5%)	5 (10.0%)	29 (32.6%)
No	15 (38.5%)	45 (90.0%)	60 (67.4%)
**Education**			
Higher Education	15 (38.5%)	15 (30.0%)	30 (33.7%)
Secondary Education	23 (59.0%)	32 (64.0%)	55 (61.8%)
Primary education	1 (2.6%)	3 (6.0%)	4 (4.5%)
**Living situation**			
Alone	7 (17.9%)	11 (22.0%)	18 (20.2%)
with others (partner, children)	32 (82.1%)	39 (78.0%)	71 (79.8%)
**Unaided phonemescore(%) at 65 dB TBI**			
Mean (SD)	1.08 (6.73)	4.33 (12.9)	2.89 (10.7)
Median [Min, Max]	0 [0, 42.0]	0 [0, 54.0]	0 [0, 54.0]
Missing	0 (0%)	1 (2.0%)	1 (1.1%)
**Unaided phonemescore(%) at 65 dB non-TBI**			
Mean (SD)	4.08 (10.5)	6.69 (14.8)	5.55 (13.1)
Median [Min, Max]	0 [0, 48.0]	0 [0, 54.0]	0 [0, 54.0]
Missing	2 (5.1%)	2 (4.0%)	4 (4.5%)
**Hearing aid use at time of referral**			
Yes	38 (97.4%)	50 (100%)	88 (98.9%)
No	1 (2.6%)	0 (0%)	1 (1.1%)
**Aided phonemescore(%) at 65 dB TBI**			
Mean (SD)	27.8 (26.1)	23.3 (22.8)	25.3 (24.3)
Median [Min, Max]	30.0 [0, 87.0]	24.8 [0, 73.0]	27.0 [0, 87.0]
**Aided phonemescore(%) at 65 dB non-TBI**			
Mean (SD)	48.1 (30.6)	42.3 (22.3)	44.9 (26.3)
Median [Min, Max]	58.5 [0, 90.0]	48.8 [0, 79.5]	51.0 [0, 90.0]

TBI = To-be-implanted (ear). * In four individuals the time between inclusion and surgery was longer than 18 months and considered outliers. Three individuals were clinically perceived to have too much residual hearing for pursuing CI. The other individual postponed surgery on personal request

## Results of the changes during waiting time

An overview table of results on all continuous outcomes on both timepoints and their differences is presented in the supplemental material (Table S2). Valid domain scores could not be calculated for all participants, either because they did not complete all questionnaire items or responded with ‘Not applicable’. The number of participants with valid domain scores is shown in column ‘N’ (in Table S2). The following section presents the domains that showed differences and their Cohen’s D and related 95% confidence intervals (95%CI).

### Impact on participation and autonomy (IPA)

Between T0 and T1 the mean scores of the total group on the IPA subdomains slightly decreased, indicating fewer difficulties in participation and autonomy. Amongst the five IPA subdomains, the difference ranged from − 0.033 for ‘autonomy indoors’ to -0.228 for ‘autonomy outdoors’. There is a slight estimated improvement in autonomy outdoors during time to surgery, with a difference of -0.228 (95%CI: -0.375 ; -0.081) and a Cohen’s D of -0.298 (95%CI: -0.493 ; -0.103). An improvement was also observed for the subdomain ‘social life and relationships’ with a difference of -0.13 (95%CI: -0.25 ; -0.006) and a Cohens D of -0.19 (95%CI: -0.37 ; -0.008).

## Work

Thirty-nine participants in the under-65 age group completed the question on paid or unpaid work at both time points. At T0 there were 30 participants (76.9%) of this group indicating to have paid or unpaid work and 9 did not (23.1%). Of these 30, 23 participants indicated to also do paid or unpaid work at T1 (76.7%). Seven of these 30 no longer stated to do paid or unpaid work at T1 (23.3%). From the 9 participants who did not do paid or unpaid work at T0, the answer remained unchanged at T1. This leads to an odds ratio (OR) for the reduction in performed paid or unpaid work during the pre-operative assessment phase of 0 (95%CI: 0–0.69). This OR also indicates that no individuals transitioned from not working at T0 to working at T1 in terms of paid or unpaid work.

Regarding paid employment, 39 participants in the under-65 age group completed the question on paid work at both time points. At T0 there were 24 participants (61.5%) of this group indicating to have paid work and 15 did not (38.5%). Of the 24 with paid employment at T0, 21 participants indicated to also do paid work at T1 (87.5%). Three of these 24 no longer stated to do paid work at T1 (12.5%). From the 15 participants who did not do work at T0, the answer remained unchanged regarding paid work status at T1. This yields an OR of 0 with a 95%-CI ranging from 0 to 2.42. This suggests that there were no observed transitions from being unemployed at T0 to being employed at T1. Additionally, the confidence interval indicates that there is no substantial change in paid employment during the waiting time in this group.

Only a small group completed items on the scales regarding work experience at T0 and T1. ‘Input/say/participation’ (*N* = 18, mean difference of 3.01 (95% CI: -3.2 ; 9.24) and Cohen’s D 0.127 (95% CI: -0.127; 0.38)), ‘relationship with coworkers’ (N = 17, mean difference of -2.83 (95% CI: -8.8 ; 3.13) and Cohen’s D -0.243 (95%CI: -0.741 ; 0.256)) and ‘need for recovery’ (N = 19, mean difference of -0.478 (95% CI: -10.96; 10) and Cohen’s D -0.017 (95%CI: -0.374 ; 0.34)). Given the confidence intervals of these effect sizes, we could not demonstrate any differences in these work experience outcomes between T0 and T1.

The List Work Capabilities (LWC) scores for the individuals who reported to perform paid work (*n* = 24) at both time points, were relatively low on the total capability set, with a mean score of 2.42 (SD 2.65) at T0 and a mean score of 3.42 (SD 2.48) at T1. These low scores at each timepoint indicate a low capability set for work suggesting lower sustainable employability in the pre-operative SMILE population. However, there appears to be an improvement in LWC total score from T0 to T1 with a mean difference of 1 (95%CI: 0.155; 1.845) and a Cohen’s D estimate of 0.388 (95%CI: 0.057 ; 0.719).

Additionally, we calculated the age-standardized unemployment rate to assess the comparison between pre-operative SMILE participants aged 18 to 75 at T0 and the general Dutch population within the same age bracket. Complete case analysis showed a standardized unemployment rate of 1.29 (95%CI: 1.17; 1.42). To assess the effect of the missing values on this result, we also analyzed the data assuming all subjects with a missing unemployment status were assumed to be employed and assuming all subjects with a missing unemployment status were assumed to be unemployed. The first scenario led to an estimated standardized unemployment rate of 1.22 and the second to an estimated standardized unemployment rate of 1.3, indicating that the missing data did not influence the finding that the estimated unemployment rate is higher for the patient group compared to the general population. This suggests that, within the SMILE population at T0, the estimated unemployment rate is likely to be at least 17% higher than what would be expected based on the general Dutch population.

### Communication profile

In the communication profile subdomains of the CPHI, a higher score indicates fewer problems experienced by the individual. Improvements were observed for four of the six CPHI subdomains during the waiting time for CI: ‘Stress and withdrawal’ ((difference = 0.216 (95% CI: 0.104 ; 0.328) (Cohens D = 0.276, 95% CI: 0.131 ; 0.42)), ‘self-acceptance’ ((difference = 0.184 (95% CI: 0.066 ; 0.302) (Cohens D = 0.205, 95% CI: 0.073 ; 0.337)), ‘maladaptive behavior’ ((difference = 0.122 (95% CI: 0.012 ; 0.233) (Cohens D = 0.166, 95% CI: 0.016 ; 0.317)), and ‘acceptance of loss’ ((difference = 0.119 (95% CI: 0.011 ; 0.228) (Cohens D = 0.166, 95%CI 0.015 ; 0.317)).

## Health-related quality of life and capability

In the generic health-related quality of life and capability scales (HUI3, EQ5D and ICECAP), only a small increase was observed between T0 and T1 in the HUI3 utility index of 0.041 (95%CI: 0.006 ; 0.075), with a Cohen’s D estimate of 0.22 (95% CI: 0.033 ; 0.407). Five subdomains changed on the disease-specific quality of life scale (NCIQ) between T0 and T1, in which higher scores indicate better results on that domain. The subdomain ‘social interaction’ showed an increase of 4.548 (95% CI: 1.5 : 7.596) and a Cohen’s D of 0.241 (95% CI: 0.078 ; 0.403). An increase was also observed for the subdomain ‘self-esteem’ with a difference of 4.956 (95% CI: 2.162 : 7.749) and a Cohen’s D of 0.268 (95% CI: 0.115 ; 0.42). The subdomains ‘activity limitations’ showed an increase of 3.109 (95%CI: 0.162 : 6.056) with a Cohen’s D of 0.162 (95% CI: 0.009 ; 0.316) and ‘speech production’ an increase of 2.794 (95% CI: 0.022 : 5.567) and a Cohen’s D of 0.156 (95% CI: 0.001 ; 0.31). However, the subdomain ‘basic sound perception’ decreased with a difference of -2.087 (95% CI: -3.907 : -0.267) and a Cohen’s D of -0.144 (95% CI: -0.269 ; -0.019).

### Third-party disability

The HII-SOP scales completed by the participant’s partner (*n* = 54) were included in the analysis. There were no observed changes in the HII-SOP subdomain or total scores between T0 and T1 during the waiting time. The total HII-SOP score was 49.26 (SD 18.05) at T0 and 47.69 (SD 17.63) at T1, suggesting that, on average, there is moderate third-party disability associated with hearing loss in this group preoperatively [21].

### Retrospective comparisons at baseline

A retrospective analysis (supplementary material) with data collected at T0 was performed to compare subgroups. Firstly it contrasted the group that received a CI with the group that did not receive a CI yet. Subsequently, within the group that eventually received a CI, it further compares those who waited longer than 6 months to those who waited shorter than 6 months. In the first comparison, there were no observed differences between the groups on the outcomes of interest. In the second comparison, the total LWC score turned out to be higher for the group with the shorter waiting time with a mean difference of 1.73 (95%CI: 0.25 ; 3.21), indicating higher sustainable employment at baseline.

## Discussion

This paper describes results on various outcomes at both pre-operative time points and the changes during the waiting time for CI surgery in individuals waiting longer than 6 months. Results show noteworthy changes during waiting time on autonomy outdoors, social life and relationships, maladaptive behavior, self-acceptance, acceptance of loss, stress and withdrawal, basic sound perception, speech production, self-esteem, activity limitations, social interaction, HUI3 utility score, ‘paid or unpaid’ work and the work capability total score. Basic sound perception and performing ‘paid or unpaid’ work showed worse outcomes at T1, the other domains in which changes were observed counterintuitively indicate an improvement in self-assessed status at the second preoperative measurement compared to the first measurement.

It could be argued that this improvement may be influenced by the provision of perspective following the decision to pursue a CI, as well as the attention and communication during clinical appointments in the CI trajectory, and the anticipation of improved hearing with a CI. Another explanation might be that some participants completed the T0 questionnaires during strict COVID-19 regulations, including lockdowns, and the T1 questionnaires after these lockdown restrictions had (partially) subsided. However, the magnitude of all the observed changes in scores was considered small. This study suggests that, in the short-term, there are no or only small consequences of longer waiting time on these participatory outcomes. It is also important to note the potential bias due to only including patients with a waiting time that exceeds 6 months. Some patients with a shorter waiting time may have been prioritized in the CI trajectory due to rapid hearing loss or declining communication abilities. If these patients had not been prioritized and had to wait longer than 6 months, their outcomes might have changed the study results.

The finding that at baseline (T0) the (age-standardized) unemployment rate of the study participants is larger than in the general Dutch population is especially relevant, as a longer duration of unemployment could also result in lower probabilities of finding employment [33, 34]. This emphasizes the need to raise awareness about the potentially eligible population for (timely) CI, aiming to mitigate the risk of unemployment. Furthermore, from an economic, psychological and emotional point of view, having to wait for elective treatment is undesirable given the prolonged period of sub-optimal health. The changes during the waiting period in our outcomes of interest suggest that longer waiting times for a CI may be deemed acceptable in the short term, for patients for whom waiting more than 6 months is acceptable due to relatively stable hearing and communication. However, some individuals referred for CI require urgent attention due to pressing needs, and such cases are likely in the group with less than 6 months of waiting time. To deliver personalized care, it is crucial to consider the heterogeneity among referred individuals in clinical practice. Further research is necessary to explore and understand for whom extended waiting periods may or may not be tolerable in both the short and long term.

The potential increase in waiting time itself is influenced by multiple factors, including demographic changes, technological advancements, political influences, and sociocultural developments that collectively could result in an escalating demand for healthcare. It is therefore also imperative to recognize that other innovations and service delivery modifications might improve efficiencies that could (in)directly impact time to implantation. Efficiencies in resource allocation might improve access to cochlear implant care without compromising waiting times. It is crucial to explore and establish standards for prioritization, non-inferiority of (service) interventions, and predictive factors for postoperative (audiological) outcomes [13, 35–38].

The results regarding changes in work status and work experience should be interpreted in light of the age categories. The analysis concerned the group aged younger than 65 years. Individuals closer to the retirement age of 67 years in the Netherlands are more likely to retire and might have stopped working regardless of their hearing loss. Nevertheless, the additional analysis on the age-standardized unemployment rate shows that individuals in the SMILE study have a higher unemployment rate compared to the general Dutch population, which emphasizes the impact of severe to profound hearing loss on employment status.

This paper does not present results on the long-term consequences of waiting for CI on measured outcomes. The postoperative results on most outcomes, like communication profile and health-related quality of life, are expected to persist over a longer period compared to the preoperative changes. Therefore, postoperative results contribute more to metrics similar to Quality Adjusted Life Years (QALYs). In decision-making contexts, there is a case for giving greater consideration to the impact of CI waiting time on long-term outcomes as opposed to short-term outcomes. Additional analysis of the SMILE data, after study completion, might provide more insights on tolerable CI waiting times for sub-groups with comparable characteristics and investigate how time to surgery affects short- and long-term outcomes and CI performance.

To the best of our knowledge, there are no other studies that investigate the changes in participatory outcomes during the waiting time for CI, although there are studies closely related. Some studies investigated the effect of the duration of (various definitions of) deafness on postoperative CI audiological performance [39]. A systematic review shows a negative correlation between the duration of auditory deprivation and postoperative CI speech perception scores [39]. The calculated correlations within two subgroups, based on more or less than 12 months of CI use, indicate a strong negative correlation in the group with less than 12 months follow-up and a moderate negative correlation in the group with more than 12 months follow-up. These results suggest that more experience with CI mitigates the negative effect of duration of deafness (considering a vast variety of definitions of deafness, including severe levels of hearing loss) on speech perception scores [39]. While the review focuses solely on audiological performance, it does suggest that the (partially) reversible nature of diminished postoperative performance may be linked to the prolonged duration of hearing loss. However, the longest duration of hearing loss likely occurs before the referral period. This underscores the need for a more systematic, regional or national approach to reduce the time of onset of CI-eligible hearing loss to implantation.

The current study has some limitations. It is an exploratory observational study, designed to closely mirror clinical practice to optimize ecological validity. The study inclusion and exclusion criteria closely resemble the adult CI eligibility criteria in clinical practice and multiple CI centers included participants. Without the randomization of study participants, however, we are compelled to examine observed changes within subjects, refraining from making assertions about approximate causality. In evaluating the findings, it is crucial to note that this study is not designed as a non-inferiority trial. In such trials, the aim is to assess whether longer waiting is not inferior to a shorter waiting period, with a pre-defined margin of equivalence for a specific outcome. Furthermore, the time of inclusion, assessment for eligibility and implantation is sequential, meaning that T0 measurements were not administered and completed at the same time period for all participants. This may have influenced (changes in) scores for some individuals, particularly since some questionnaires were completed during COVID-19 regulations, the strictness of these regulations at the time of survey completion could have differentially affected participatory outcome scores for individuals at T0 and T1. Also, the sensitivity of the measurement instruments used should be considered since some questionnaires were not validated in this specific population and might not be responsive and sensitive enough to detect subtle changes in health status. Participants might also have experienced a response shift regarding their health in anticipation of CI surgery.

In conclusion, this study showed an impact of hearing loss in individuals referred for CI in comparison to the general population regarding generically interpretable outcomes such as, amongst others, the HUI3 and unemployment rate. We observed no or only small changes in participatory outcomes during the CI waiting time. Extended CI waiting times for CI may have long-term consequences on similar outcomes, and there is a need for further exploration in this regard. Future studies should also aim to identify subgroups and might consider creating specific minimum standards for the quality and accessibility of CI care.

## Electronic supplementary material

Below is the link to the electronic supplementary material.

Supplementary Material 1

## Data Availability

Data is available in collaboration and upon reasonable request, after study completion.

## Code Availability

Currently not available. Available on reasonable request.
