# Supplementary material for: Changes in participatory and societal outcomes during the waiting period for cochlear implantation – an observational study
Source: Eur Arch Otorhinolaryngol. 2024 Sep 26;282(2):753–64. doi: 10.1007/s00405-024-08981-7 (PMC11805756; doi:10.1007/s00405-024-08981-7)
Supplement: Supplementary file 1 — Supplementary Material 1 [file 405_2024_8981_MOESM1_ESM.docx]

Supplemental Material

Title: Changes in participatory and societal outcomes during the waiting period for cochlear implantation – an observational study.

Authors: H.G.B. Nijmeijer1,2, N. Philpott1,2, GJ van der Wilt2,3, A.R.T Donders3, E. George4, R. Boerboom5, J.H.M. Frijns6,7,8, M. Kaandorp9, W.J. Huinck1,2, E.A.M. Mylanus1,2

Affiliations:

1 Department for Otorhinolaryngology, Radboud university medical center, Nijmegen, The Netherlands

2 Donders Institute for Brain, Cognition and Behaviour, Radboud University, Nijmegen, The Netherlands

3 Department for Health Evidence, Radboud university medical center, Nijmegen, The Netherlands

4. Department of ENT/Audiology, School for Mental Health and Neuroscience, Maastricht University Medical Centre, Maastricht, The Netherlands

5. Department of Otorhinolaryngology, Head and Neck Surgery, University Medical Center Utrecht, Utrecht University, Utrecht, The Netherlands

6. Department of Otorhinolaryngology, Leiden University Medical Center, Leiden, The Netherlands

7 Leiden Institute for Brain and Cognition, Leiden, The Netherlands

8 Department of Bioelectronics, Delft University of Technology, Delft, The Netherlands

9 Amsterdam University Medical Center location Vrije Universiteit, Department of Otolaryngology - Head and Neck Surgery, section Ear & Hearing, Amsterdam Public Health research institute, Amsterdam, The Netherlands

# Supplemental Material – Table of content

[Supplemental Material – Table of content 2](#_Toc156469367)

[Supplemental Material – Extended baseline (T0) characteristics table of the individuals who experienced a waiting time for surgery longer than 6 months and (partially) completed T0 and T1 surveys. 3](#_Toc156469368)

[Supplemental Material – Results of changes during waiting time 7](#_Toc156469369)

[Supplemental Material – Retrospective T0 difference between individuals with and without a surgery date. 11](#_Toc156469370)

[Supplemental Material - Retrospective T0 difference between individuals with a time between study inclusion and surgery of more or less than 6 months. 19](#_Toc156469371)

# Supplemental Material – Extended baseline (T0) characteristics table of the individuals who experienced a waiting time for surgery longer than 6 months and (partially) completed T0 and T1 surveys.

Table s1. Extended baseline (T0) characteristics of SMILE participants (partially) completed T0 and T1 surveys.

|  | Under 65 (N=39) | Over 65 (N=50) | Total at T0 (N=89) |
| --- | --- | --- | --- |
| Age at inclusion (years) |  |  |  |
| Mean (SD) | 54.7 (8.97) | 72.4 (4.75) | 64.7 (11.2) |
| Median [Min, Max] | 58.0 [31.0, 64.0] | 72.0 [65.0, 85.0] | 67.0 [31.0, 85.0] |
| Sex |  |  |  |
| Male | 17 (43.6%) | 28 (56.0%) | 45 (50.6%) |
| Female | 22 (56.4%) | 22 (44.0%) | 44 (49.4%) |
| Time between inclusion and surgery (months) |  |  |  |
| Mean (SD) | 11.4 (5.44) | 11.2 (3.07) | 11.3 (4.25) |
| Median [Min, Max] | 10.6 [6.40, 33.7] | 11.5 [6.10, 17.9] | 11.0 [6.10, 33.7] |
| Age of noticing hearing loss (years) |  |  |  |
| Mean (SD) | 28.1 (16.2) | 46.2 (17.6) | 38.8 (19.1) |
| Median [Min, Max] | 30.0 [0, 54.0] | 50.0 [0, 79.0] | 41.0 [0, 79.0] |
| Missing | 5 (12.8%) | 1 (2.0%) | 6 (6.7%) |
| Duration of hearing loss (years) |  |  |  |
| Mean (SD) | 26.4 (13.2) | 26.2 (18.0) | 26.3 (16.1) |
| Median [Min, Max] | 22.5 [9.00, 61.0] | 20.0 [2.00, 80.0] | 21.0 [2.00, 80.0] |
| Missing | 5 (12.8%) | 1 (2.0%) | 6 (6.7%) |
| Category etiology of hearing loss |  |  |  |
| Acquired | 6 (15.4%) | 10 (20.0%) | 16 (18.0%) |
| Not-syndromal | 10 (25.6%) | 9 (18.0%) | 19 (21.3%) |
| Syndromal | 4 (10.3%) | 2 (4.0%) | 6 (6.7%) |
| Unknown | 19 (48.7%) | 29 (58.0%) | 48 (53.9%) |
| Paid or unpaid Work |  |  |  |
| Yes | 30 (76.9%) | 26 (52.0%) | 56 (62.9%) |
| No | 9 (23.1%) | 24 (48.0%) | 33 (37.1%) |
| Paid Work |  |  |  |
| Yes | 24 (61.5%) | 5 (10.0%) | 29 (32.6%) |
| No | 15 (38.5%) | 45 (90.0%) | 60 (67.4%) |
| Education |  |  |  |
| Higher Education | 15 (38.5%) | 15 (30.0%) | 30 (33.7%) |
| Secondary Education | 23 (59.0%) | 32 (64.0%) | 55 (61.8%) |
| Primary education | 1 (2.6%) | 3 (6.0%) | 4 (4.5%) |
| Living situation |  |  |  |
| Alone | 7 (17.9%) | 11 (22.0%) | 18 (20.2%) |
| with others (partner, children) | 32 (82.1%) | 39 (78.0%) | 71 (79.8%) |
| Unaided phonemescore(%) at 65 dB TBI |  |  |  |
| Mean (SD) | 1.08 (6.73) | 4.33 (12.9) | 2.89 (10.7) |
| Median [Min, Max] | 0 [0, 42.0] | 0 [0, 54.0] | 0 [0, 54.0] |
| Missing | 0 (0%) | 1 (2.0%) | 1 (1.1%) |
| Unaided phonemescore(%) at 65 dB non-TBI |  |  |  |
| Mean (SD) | 4.08 (10.5) | 6.69 (14.8) | 5.55 (13.1) |
| Median [Min, Max] | 0 [0, 48.0] | 0 [0, 54.0] | 0 [0, 54.0] |
| Missing | 2 (5.1%) | 2 (4.0%) | 4 (4.5%) |
| Unaided phonemescore(%) at 75 dB TBI |  |  |  |
| Mean (SD) | 4.08 (10.8) | 7.44 (15.7) | 5.97 (13.8) |
| Median [Min, Max] | 0 [0, 54.0] | 0 [0, 51.0] | 0 [0, 54.0] |
| Unaided phonemescore(%) at 75 dB non-TBI |  |  |  |
| Mean (SD) | 14.6 (21.2) | 14.4 (21.2) | 14.5 (21.1) |
| Median [Min, Max] | 0 [0, 66.0] | 0 [0, 70.0] | 0 [0, 70.0] |
| Missing | 1 (2.6%) | 1 (2.0%) | 2 (2.2%) |
| Unaided phonemescore(%) at 85 dB TBI |  |  |  |
| Mean (SD) | 10.5 (17.1) | 11.0 (18.1) | 10.8 (17.6) |
| Median [Min, Max] | 0 [0, 57.0] | 0 [0, 57.0] | 0 [0, 57.0] |
| Unaided phonemescore(%) at 85 dB non-TBI |  |  |  |
| Mean (SD) | 27.6 (27.2) | 22.1 (25.1) | 24.5 (26.0) |
| Median [Min, Max] | 24.0 [0, 84.0] | 7.50 [0, 75.0] | 18.0 [0, 84.0] |
| Hearing aid use at time of referral |  |  |  |
| Yes | 38 (97.4%) | 50 (100%) | 88 (98.9%) |
| No | 1 (2.6%) | 0 (0%) | 1 (1.1%) |
| Aided phonemescore(%) at 55 dB TBI |  |  |  |
| Mean (SD) | 12.3 (17.5) | 9.80 (14.9) | 11.0 (16.1) |
| Median [Min, Max] | 0 [0, 66.0] | 0 [0, 52.0] | 0 [0, 66.0] |
| Missing | 0 (0%) | 5 (10.0%) | 5 (5.6%) |
| Aided phonemescore(%) at 55 dB non-TBI |  |  |  |
| Mean (SD) | 25.3 (23.1) | 19.4 (19.0) | 22.1 (21.1) |
| Median [Min, Max] | 24.0 [0, 69.0] | 18.0 [0, 57.0] | 18.0 [0, 69.0] |
| Missing | 2 (5.1%) | 7 (14.0%) | 9 (10.1%) |
| Aided phonemescore(%) at 65 dB TBI |  |  |  |
| Mean (SD) | 27.8 (26.1) | 23.3 (22.8) | 25.3 (24.3) |
| Median [Min, Max] | 30.0 [0, 87.0] | 24.8 [0, 73.0] | 27.0 [0, 87.0] |
| Aided phonemescore(%) at 65 dB non-TBI |  |  |  |
| Mean (SD) | 48.1 (30.6) | 42.3 (22.3) | 44.9 (26.3) |
| Median [Min, Max] | 58.5 [0, 90.0] | 48.8 [0, 79.5] | 51.0 [0, 90.0] |
| Aided phonemescore(%) at 75 dB TBI |  |  |  |
| Mean (SD) | 35.0 (27.6) | 30.3 (26.5) | 32.3 (26.9) |
| Median [Min, Max] | 42.0 [0, 96.0] | 37.5 [0, 88.0] | 39.0 [0, 96.0] |
| Aided phonemescore(%) at 75 dB non-TBI |  |  |  |
| Mean (SD) | 52.8 (32.0) | 55.7 (24.0) | 54.4 (27.6) |
| Median [Min, Max] | 63.0 [0, 96.0] | 63.0 [0, 88.5] | 63.0 [0, 96.0] |

# Supplemental Material – Results of changes during waiting time

Table s2. overview table of results on all continuous outcomes on both timepoints and their differences are presented in the supplemental material

| Names | N | T0 mean (SD) | T0 median (ranges) | T1 mean (SD) | T1 median (ranges) | Difference (95% CI) | p-value* | Correlation (95% CI) | Cohen's D (95% CI) | Cohens D rank |
| --- | --- | --- | --- | --- | --- | --- | --- | --- | --- | --- |
| (IPA) Autonomy indoors | 87 | 0.414 (0.518) | 0.143 (0 - 2) | 0.381 (0.474) | 0 (0 - 1.714) | -0.033 (-0.137 : 0.071) | 0.58802 | 0.52 (0.347 ; 0.658) | -0.066 (-0.274 ; 0.142) | 21.0 |
| (IPA) Autonomy outdoors | 87 | 1.577 (0.799) | 1.6 (0 - 3.2) | 1.349 (0.722) | 1.4 (0 - 3) | -0.228 (-0.375 : -0.081) | 0.00187 | 0.593 (0.437 ; 0.714) | -0.298 (-0.493 ; -0.103) | 2.0 |
| (IPA) Family role | 87 | 0.89 (0.788) | 1 (0 - 3) | 0.854 (0.698) | 0.857 (0 - 2.571) | -0.036 (-0.148 : 0.076) | 0.37294 | 0.758 (0.651 ; 0.835) | -0.048 (-0.195 ; 0.099) | 23.0 |
| (IPA) Social life and relationships | 87 | 1.629 (0.737) | 1.571 (0 - 3.429) | 1.499 (0.635) | 1.429 (0.286 - 3.286) | -0.13 (-0.253 : -0.006) | 0.06063 | 0.651 (0.511 ; 0.758) | -0.187 (-0.365 ; -0.008) | 9.0 |
| (IPA) Work and Education | 33 | 2.308 (0.922) | 2.333 (1 - 4.333) | 2.197 (0.978) | 2 (0.333 - 3.667) | -0.111 (-0.286 : 0.063) | 0.21027 | 0.867 (0.746 ; 0.933) | -0.116 (-0.296 ; 0.064) | 17.0 |
| (CPHI) Maladaptive Behavior | 87 | 3.431 (0.707) | 3.5 (2.125 - 5) | 3.553 (0.757) | 3.75 (1.75 - 5) | 0.122 (0.012 : 0.233) | 0.03173 | 0.751 (0.642 ; 0.83) | 0.166 (0.016 ; 0.317) | 11.5 |
| (CPHI) Verbal Strategies | 87 | 3.246 (0.81) | 3.25 (1.75 - 5) | 3.253 (0.851) | 3.125 (1.25 - 5) | 0.007 (-0.119 : 0.134) | 0.73330 | 0.745 (0.634 ; 0.826) | 0.009 (-0.142 ; 0.16) | 26.5 |
| (CPHI) Nonverbal Strategies | 87 | 3.897 (0.739) | 4 (1.429 - 5) | 3.883 (0.802) | 4 (1.857 - 5) | -0.013 (-0.146 : 0.119) | 0.37955 | 0.677 (0.544 ; 0.777) | -0.017 (-0.187 ; 0.153) | 24.5 |
| (CPHI) Self-Acceptance | 87 | 3.801 (0.912) | 4 (1.167 - 5) | 3.985 (0.875) | 4.167 (1.333 - 5) | 0.184 (0.066 : 0.302) | 0.00177 | 0.81 (0.722 ; 0.872) | 0.205 (0.073 ; 0.337) | 8.0 |
| (CPHI) Acceptance of Loss | 87 | 3.624 (0.736) | 3.75 (1.75 - 5) | 3.743 (0.695) | 3.875 (1.875 - 4.875) | 0.119 (0.011 : 0.228) | 0.02605 | 0.749 (0.639 ; 0.829) | 0.166 (0.015 ; 0.317) | 11.5 |
| (CPHI) Stress, and Withdrawal | 87 | 2.498 (0.764) | 2.533 (1.133 - 4.533) | 2.714 (0.8) | 2.667 (1 - 4.8) | 0.216 (0.104 : 0.328) | 0.00014 | 0.776 (0.675 ; 0.848) | 0.276 (0.131 ; 0.42) | 3.0 |
| (NCIQ) Basic sound perception | 82 | 27.043 (14.677) | 25 (0 - 72.5) | 24.956 (14.251) | 22.5 (0 - 72.5) | -2.087 (-3.907 : -0.267) | 0.03917 | 0.836 (0.757 ; 0.892) | -0.144 (-0.269 ; -0.019) | 15.0 |
| (NCIQ) Advanced sound perception | 82 | 31.069 (15.446) | 30 (0 - 72.5) | 32.297 (16.361) | 30 (0 - 77.5) | 1.228 (-0.963 : 3.419) | 0.29122 | 0.805 (0.712 ; 0.87) | 0.077 (-0.059 ; 0.213) | 20.0 |
| (NCIQ) Speech production | 80 | 71.088 (17.187) | 72.5 (25 - 100) | 73.882 (18.484) | 75 (22.5 - 100) | 2.794 (0.022 : 5.567) | 0.08062 | 0.758 (0.646 ; 0.838) | 0.156 (0.001 ; 0.31) | 14.0 |
| (NCIQ) Self esteem | 82 | 50.093 (18.358) | 50 (8.333 - 95) | 55.049 (18.669) | 53.889 (12.5 - 95) | 4.956 (2.162 : 7.749) | 0.00081 | 0.764 (0.656 ; 0.842) | 0.268 (0.115 ; 0.42) | 4.0 |
| (NCIQ) Activity limitations | 80 | 48.428 (17.959) | 47.222 (10 - 82.5) | 51.536 (19.993) | 50 (7.5 - 97.222) | 3.109 (0.162 : 6.056) | 0.05672 | 0.762 (0.651 ; 0.841) | 0.162 (0.009 ; 0.316) | 13.0 |
| (NCIQ) Social interaction | 81 | 48.329 (18.231) | 45 (12.5 - 92.5) | 52.877 (19.427) | 53.571 (11.111 - 91.667) | 4.548 (1.5 : 7.596) | 0.00773 | 0.734 (0.614 ; 0.821) | 0.241 (0.078 ; 0.403) | 6.0 |
| HUI3 Score | 87 | 0.459 (0.18) | 0.439 (0.04 - 0.849) | 0.5 (0.19) | 0.514 (0.001 - 0.849) | 0.041 (0.006 : 0.075) | 0.02823 | 0.619 (0.47 ; 0.734) | 0.22 (0.033 ; 0.407) | 7.0 |
| EQ5D VAS score | 82 | 79.976 (13.49) | 80 (40 - 100) | 79.951 (12.774) | 80 (30 - 100) | -0.024 (-3.002 : 2.953) | 0.85121 | 0.469 (0.28 ; 0.622) | -0.002 (-0.227 ; 0.223) | 28.0 |
| EQ5D Value | 82 | 0.851 (0.146) | 0.885 (0.383 - 1) | 0.861 (0.145) | 0.885 (0.235 - 1) | 0.009 (-0.017 : 0.035) | 0.22384 | 0.675 (0.536 ; 0.778) | 0.063 (-0.113 ; 0.239) | 22.0 |
| ICECAP-A Value | 84 | 0.85 (0.115) | 0.88 (0.369 - 1) | 0.851 (0.124) | 0.881 (0.411 - 1) | 0.001 (-0.022 : 0.024) | 1.00000 | 0.602 (0.446 ; 0.723) | 0.009 (-0.184 ; 0.201) | 26.5 |
| (HIISOP) Communication strategy | 54 | 18.796 (3.658) | 20 (5 - 25) | 18.796 (3.212) | 20 (10 - 25) | 0 (-1.044 : 1.044) | 0.96374 | 0.386 (0.132 ; 0.593) | 0 (-0.299 ; 0.299) | 29.0 |
| (HIISOP) Relationship and emotions | 54 | 22.731 (12.355) | 21.25 (0 - 52.5) | 20.694 (11.71) | 20 (0 - 50) | -2.037 (-4.356 : 0.282) | 0.06366 | 0.752 (0.606 ; 0.849) | -0.169 (-0.36 ; 0.023) | 10.0 |
| (HIISOP) Social impact | 54 | 7.731 (5.37) | 7.5 (0 - 20) | 8.194 (5.417) | 7.5 (0 - 17.5) | 0.463 (-0.731 : 1.657) | 0.42973 | 0.671 (0.492 ; 0.796) | 0.086 (-0.133 ; 0.305) | 19.0 |
| (HIISOP) Total | 54 | 49.259 (18.045) | 46.25 (15 - 90) | 47.685 (17.63) | 45 (15 - 90) | -1.574 (-5.124 : 1.976) | 0.33834 | 0.734 (0.581 ; 0.837) | -0.088 (-0.285 ; 0.109) | 18.0 |
| (QEEW) Need for recovery | 19 | 40.67 (29.124) | 36.364 (9.091 - 81.818) | 40.191 (27.326) | 36.364 (9.091 - 81.818) | -0.478 (-10.957 : 10) | 0.46252 | 0.705 (0.369 ; 0.878) | -0.017 (-0.374 ; 0.34) | 24.5 |
| (QEEW) Relationships with colleagues | 17 | 52.505 (10.737) | 55.556 (33.333 - 70.37) | 49.673 (12.427) | 51.852 (22.222 - 66.667) | -2.832 (-8.798 : 3.134) | 0.64870 | 0.506 (0.034 ; 0.794) | -0.243 (-0.741 ; 0.256) | 5.0 |
| (QEEW) Input/say/participaton | 18 | 52.546 (22.962) | 50 (12.5 - 91.667) | 55.556 (24.338) | 54.167 (12.5 - 100) | 3.009 (-3.219 : 9.238) | 0.34849 | 0.861 (0.66 ; 0.947) | 0.127 (-0.127 ; 0.38) | 16.0 |
| (LWC)Total score | 24 | 2.417 (2.653) | 1 (0 - 7) | 3.417 (2.483) | 4 (0 - 7) | 1 (0.155 : 1.845) | 0.03408 | 0.699 (0.411 ; 0.86) | 0.388 (0.057 ; 0.719) | 1.0 |

This table shows the results for SMILE participants with valid scores for calculating the subdomains on both T0 and T1. Number of individuals with valid scores on both time points are indicated by N.
* p-values from Wilcoxon signed rank test with two sided continuity correction are not corrected for multiple comparisons and should be interpreted with caution.

# Supplemental Material – Retrospective T0 difference between individuals with and without a surgery date.

Table s3. Base line characteristics (T0) for participants with and without (No) surgery (date)

|  | Surgery date group (N=150) | No Surgery date group (N=66) | Total at T0 (N=216) |
| --- | --- | --- | --- |
| Age at inclusion (years) |  |  |  |
| Mean (SD) | 64.8 (12.2) | 63.9 (13.5) | 64.5 (12.6) |
| Median [Min, Max] | 67.5 [18.0, 88.0] | 66.5 [29.0, 82.0] | 67.0 [18.0, 88.0] |
| Age category |  |  |  |
| Over 65 | 88 (58.7%) | 41 (62.1%) | 129 (59.7%) |
| Under 65 | 62 (41.3%) | 25 (37.9%) | 87 (40.3%) |
| Sex |  |  |  |
| Male | 84 (56.0%) | 30 (45.5%) | 114 (52.8%) |
| Female | 66 (44.0%) | 36 (54.5%) | 102 (47.2%) |
| Time between inclusion and surgery (months) |  |  |  |
| Mean (SD) | 8.98 (4.73) | NA (NA) | 8.98 (4.73) |
| Median [Min, Max] | 8.50 [1.00, 33.7] | NA [NA, NA] | 8.50 [1.00, 33.7] |
| Missing | 0 (0%) | 66 (100%) | 66 (30.6%) |
| Age of noticing hearing loss (years) |  |  |  |
| Mean (SD) | 39.6 (18.2) | 37.2 (20.9) | 38.9 (18.9) |
| Median [Min, Max] | 42.0 [0, 79.0] | 40.0 [0, 77.0] | 40.0 [0, 79.0] |
| Missing | 7 (4.7%) | 11 (16.7%) | 18 (8.3%) |
| Duration of hearing loss (years) |  |  |  |
| Mean (SD) | 25.3 (15.0) | 26.7 (15.9) | 25.7 (15.2) |
| Median [Min, Max] | 20.0 [2.00, 80.0] | 25.0 [1.00, 67.0] | 22.0 [1.00, 80.0] |
| Missing | 7 (4.7%) | 11 (16.7%) | 18 (8.3%) |
| Category etiology of hearing loss |  |  |  |
| Acquired | 25 (16.7%) | 16 (24.2%) | 41 (19.0%) |
| Not-syndromal | 29 (19.3%) | 7 (10.6%) | 36 (16.7%) |
| Other | 2 (1.3%) | 2 (3.0%) | 4 (1.9%) |
| Syndromal | 6 (4.0%) | 1 (1.5%) | 7 (3.2%) |
| Unknown | 88 (58.7%) | 37 (56.1%) | 125 (57.9%) |
| Missing | 0 (0%) | 3 (4.5%) | 3 (1.4%) |
| Paid or unpaid Work |  |  |  |
| Yes | 86 (57.3%) | 31 (47.0%) | 117 (54.2%) |
| No | 63 (42.0%) | 31 (47.0%) | 94 (43.5%) |
| Missing | 1 (0.7%) | 4 (6.1%) | 5 (2.3%) |
| Paid Work |  |  |  |
| Yes | 49 (32.7%) | 19 (28.8%) | 68 (31.5%) |
| No | 99 (66.0%) | 41 (62.1%) | 140 (64.8%) |
| Missing | 2 (1.3%) | 6 (9.1%) | 8 (3.7%) |
| Education |  |  |  |
| Higher Education | 58 (38.7%) | 20 (30.3%) | 78 (36.1%) |
| Secondary Education | 85 (56.7%) | 36 (54.5%) | 121 (56.0%) |
| Primary education | 6 (4.0%) | 3 (4.5%) | 9 (4.2%) |
| Missing | 1 (0.7%) | 7 (10.6%) | 8 (3.7%) |
| Living situation |  |  |  |
| Alone | 26 (17.3%) | 14 (21.2%) | 40 (18.5%) |
| with others (partner, children) | 123 (82.0%) | 45 (68.2%) | 168 (77.8%) |
| Missing | 1 (0.7%) | 7 (10.6%) | 8 (3.7%) |
| Unaided phonemescore(%) at 65 dB TBI |  |  |  |
| Mean (SD) | 3.31 (11.8) | NA (NA) | 3.31 (11.8) |
| Median [Min, Max] | 0 [0, 76.0] | NA [NA, NA] | 0 [0, 76.0] |
| Missing | 3 (2.0%) | 66 (100%) | 69 (31.9%) |
| Unaided phonemescore(%) at 65 dB non-TBI |  |  |  |
| Mean (SD) | 5.53 (13.9) | NA (NA) | 5.53 (13.9) |
| Median [Min, Max] | 0 [0, 79.0] | NA [NA, NA] | 0 [0, 79.0] |
| Missing | 8 (5.3%) | 66 (100%) | 74 (34.3%) |
| Unaided phonemescore(%) at 75 dB TBI |  |  |  |
| Mean (SD) | 6.66 (15.5) | NA (NA) | 6.66 (15.5) |
| Median [Min, Max] | 0 [0, 85.0] | NA [NA, NA] | 0 [0, 85.0] |
| Missing | 1 (0.7%) | 66 (100%) | 67 (31.0%) |
| Unaided phonemescore(%) at 75 dB non-TBI |  |  |  |
| Mean (SD) | 13.4 (20.6) | NA (NA) | 13.4 (20.6) |
| Median [Min, Max] | 0 [0, 88.0] | NA [NA, NA] | 0 [0, 88.0] |
| Missing | 5 (3.3%) | 66 (100%) | 71 (32.9%) |
| Unaided phonemescore(%) at 85 dB TBI |  |  |  |
| Mean (SD) | 11.3 (19.1) | NA (NA) | 11.3 (19.1) |
| Median [Min, Max] | 0 [0, 73.0] | NA [NA, NA] | 0 [0, 73.0] |
| Missing | 0 (0%) | 66 (100%) | 66 (30.6%) |
| Unaided phonemescore(%) at 85 dB non-TBI |  |  |  |
| Mean (SD) | 24.5 (25.9) | NA (NA) | 24.5 (25.9) |
| Median [Min, Max] | 18.0 [0, 84.0] | NA [NA, NA] | 18.0 [0, 84.0] |
| Missing | 0 (0%) | 66 (100%) | 66 (30.6%) |
| Hearing aid use |  |  |  |
| Yes | 147 (98.0%) | 58 (87.9%) | 205 (94.9%) |
| No | 3 (2.0%) | 5 (7.6%) | 8 (3.7%) |
| Missing | 0 (0%) | 3 (4.5%) | 3 (1.4%) |
| Aided phonemescore(%) at 55 dB TBI |  |  |  |
| Mean (SD) | 11.0 (16.7) | NA (NA) | 11.0 (16.7) |
| Median [Min, Max] | 0 [0, 73.0] | NA [NA, NA] | 0 [0, 73.0] |
| Missing | 11 (7.3%) | 66 (100%) | 77 (35.6%) |
| Aided phonemescore(%) at 55 dB non-TBI |  |  |  |
| Mean (SD) | 22.8 (21.6) | NA (NA) | 22.8 (21.6) |
| Median [Min, Max] | 19.0 [0, 76.0] | NA [NA, NA] | 19.0 [0, 76.0] |
| Missing | 19 (12.7%) | 66 (100%) | 85 (39.4%) |
| Aided phonemescore(%) at 65 dB TBI |  |  |  |
| Mean (SD) | 25.6 (24.3) | NA (NA) | 25.6 (24.3) |
| Median [Min, Max] | 24.5 [0, 87.0] | NA [NA, NA] | 24.5 [0, 87.0] |
| Missing | 0 (0%) | 66 (100%) | 66 (30.6%) |
| Aided phonemescore(%) at 65 dB non-TBI |  |  |  |
| Mean (SD) | 45.7 (26.6) | NA (NA) | 45.7 (26.6) |
| Median [Min, Max] | 52.0 [0, 91.0] | NA [NA, NA] | 52.0 [0, 91.0] |
| Missing | 0 (0%) | 66 (100%) | 66 (30.6%) |
| Aided phonemescore(%) at 75 dB TBI |  |  |  |
| Mean (SD) | 32.4 (28.1) | NA (NA) | 32.4 (28.1) |
| Median [Min, Max] | 36.0 [0, 96.0] | NA [NA, NA] | 36.0 [0, 96.0] |
| Missing | 0 (0%) | 66 (100%) | 66 (30.6%) |
| Aided phonemescore(%) at 75 dB non-TBI |  |  |  |
| Mean (SD) | 54.6 (27.2) | NA (NA) | 54.6 (27.2) |
| Median [Min, Max] | 61.8 [0, 97.0] | NA [NA, NA] | 61.8 [0, 97.0] |
| Missing | 0 (0%) | 66 (100%) | 66 (30.6%) |

Table s4. Baseline (T0) outcome scores for participants with valid scores in the group with and without (No) a surgery date and the differences between the groups.

| Names | N (Surgery date group) | Surgery date group mean (SD) | Surgery date group median (ranges) | N (No Surgery date group) | No Surgery date group mean (SD) | No Surgery date group. median (ranges) | Difference (95% CI) | p-value* | Cohen's D (95% CI) | Cohens D rank |
| --- | --- | --- | --- | --- | --- | --- | --- | --- | --- | --- |
| (IPA) Autonomy indoors | 149 | 0.406 (0.558) | 0.143 (0 - 3) | 62 | 0.514 (0.625) | 0.286 (0 - 2.429) | 0.108 (-0.073 : 0.29) | 0.32777 | 0.187 (-0.111 ; 0.486) | 9 |
| (IPA) Autonomy outdoors | 149 | 1.447 (0.805) | 1.4 (0 - 4) | 62 | 1.5 (0.848) | 1.4 (0 - 3.4) | 0.053 (-0.197 : 0.303) | 0.70123 | 0.065 (-0.233 ; 0.363) | 24 |
| (IPA) Family role | 149 | 0.817 (0.799) | 0.714 (0 - 3.714) | 62 | 0.903 (0.761) | 1 (0 - 2.857) | 0.086 (-0.145 : 0.318) | 0.24971 | 0.11 (-0.189 ; 0.408) | 15 |
| (IPA) Social life and relationships | 149 | 1.517 (0.736) | 1.429 (0 - 3.857) | 62 | 1.668 (0.753) | 1.5 (0.286 - 3.286) | 0.151 (-0.073 : 0.375) | 0.22597 | 0.204 (-0.094 ; 0.503) | 8 |
| (IPA) Work and Education | 66 | 2.242 (1.007) | 2.333 (0.167 - 4.333) | 27 | 2.226 (0.899) | 2.167 (0.667 - 4) | -0.016 (-0.443 : 0.411) | 0.92571 | -0.016 (-0.47 ; 0.437) | 28 |
| (CPHI) Maladaptive Behavior | 149 | 3.42 (0.752) | 3.5 (1.75 - 5) | 60 | 3.541 (0.803) | 3.625 (1.75 - 4.875) | 0.12 (-0.119 : 0.36) | 0.19101 | 0.157 (-0.145 ; 0.459) | 10 |
| (CPHI) Verbal Strategies | 149 | 3.23 (0.801) | 3.25 (1.25 - 5) | 60 | 3.308 (0.771) | 3.375 (1.375 - 5) | 0.078 (-0.158 : 0.314) | 0.38109 | 0.098 (-0.204 ; 0.4) | 18 |
| (CPHI) Nonverbal Strategies | 149 | 3.855 (0.753) | 4 (1.429 - 5) | 60 | 3.81 (0.856) | 3.857 (1.429 - 5) | -0.045 (-0.296 : 0.206) | 0.89021 | -0.057 (-0.359 ; 0.244) | 25 |
| (CPHI) Self-Acceptance | 149 | 3.761 (0.894) | 3.833 (1.167 - 5) | 59 | 3.698 (1.072) | 4 (1 - 5) | -0.063 (-0.376 : 0.25) | 0.95608 | -0.066 (-0.37 ; 0.237) | 23 |
| (CPHI) Acceptance of Loss | 149 | 3.622 (0.744) | 3.75 (1.25 - 5) | 59 | 3.558 (0.768) | 3.875 (1.25 - 5) | -0.065 (-0.297 : 0.168) | 0.77719 | -0.086 (-0.39 ; 0.217) | 20 |
| (CPHI) Stress, and Withdrawal | 149 | 2.483 (0.761) | 2.467 (1 - 5) | 59 | 2.584 (0.785) | 2.533 (1 - 4.267) | 0.101 (-0.136 : 0.338) | 0.40453 | 0.132 (-0.172 ; 0.435) | 12 |
| (NCIQ) Basic sound perception | 147 | 27.245 (14.945) | 25 (0 - 72.5) | 58 | 28.537 (18.316) | 23.75 (5 - 90) | 1.292 (-4.078 : 6.662) | 0.86182 | 0.081 (-0.225 ; 0.387) | 21 |
| (NCIQ) Advanced sound perception | 147 | 32.012 (16.255) | 30 (0 - 90) | 58 | 35.884 (18.928) | 35 (2.5 - 72.5) | 3.871 (-1.737 : 9.48) | 0.18736 | 0.227 (-0.079 ; 0.534) | 6 |
| (NCIQ) Speech production | 144 | 71.832 (17.95) | 72.5 (12.5 - 100) | 55 | 73.038 (18.511) | 75 (27.5 - 100) | 1.206 (-4.571 : 6.983) | 0.62582 | 0.067 (-0.246 ; 0.379) | 22 |
| (NCIQ) Self esteem | 147 | 51.204 (18.348) | 50 (8.333 - 97.5) | 58 | 55.19 (18.594) | 56.528 (7.5 - 90) | 3.986 (-1.711 : 9.682) | 0.14237 | 0.216 (-0.09 ; 0.523) | 7 |
| (NCIQ) Activity limitations | 146 | 48.477 (18.565) | 47.361 (2.5 - 95) | 56 | 48.924 (20.825) | 50 (2.5 - 90) | 0.447 (-5.868 : 6.762) | 0.78381 | 0.023 (-0.287 ; 0.333) | 27 |
| (NCIQ) Social interaction | 147 | 49.424 (17.615) | 50 (7.143 - 92.5) | 57 | 51.196 (20.141) | 55 (0 - 86.111) | 1.772 (-4.262 : 7.806) | 0.32729 | 0.097 (-0.211 ; 0.404) | 19 |
| HUI3 Score | 149 | 0.462 (0.197) | 0.481 (-0.228 - 0.849) | 60 | 0.434 (0.262) | 0.448 (-0.068 - 0.931) | -0.028 (-0.103 : 0.046) | 0.50684 | -0.129 (-0.431 ; 0.172) | 13 |
| EQ5D VAS score | 146 | 79.479 (13.916) | 80 (30 - 100) | 59 | 74.797 (16.905) | 80 (20 - 100) | -4.683 (-9.617 : 0.251) | 0.09071 | -0.316 (-0.621 ; -0.01) | 3 |
| EQ5D Value | 146 | 0.84 (0.168) | 0.883 (0.012 - 1) | 59 | 0.79 (0.204) | 0.817 (-0.058 - 1) | -0.051 (-0.11 : 0.009) | 0.06905 | -0.285 (-0.59 ; 0.021) | 5 |
| ICECAP-A Value | 147 | 0.851 (0.126) | 0.881 (0.364 - 1) | 59 | 0.831 (0.144) | 0.876 (0.371 - 1) | -0.02 (-0.063 : 0.022) | 0.37955 | -0.156 (-0.46 ; 0.149) | 11 |
| (HIISOP) Communication strategy | 115 | 19.348 (3.629) | 20 (5 - 25) | 36 | 17.917 (4.8) | 18.75 (7.5 - 25) | -1.431 (-3.177 : 0.315) | 0.11126 | -0.364 (-0.743 ; 0.016) | 2 |
| (HIISOP) Relationship and emotions | 115 | 22.848 (12.363) | 22.5 (0 - 55) | 36 | 22.708 (13.672) | 25 (2.5 - 50) | -0.139 (-5.259 : 4.98) | 0.95636 | -0.011 (-0.388 ; 0.366) | 29 |
| (HIISOP) Social impact | 115 | 7.978 (5.589) | 7.5 (0 - 20) | 36 | 7.361 (6.351) | 5 (0 - 20) | -0.617 (-2.984 : 1.749) | 0.46172 | -0.107 (-0.484 ; 0.271) | 16 |
| (HIISOP) Total | 115 | 50.174 (18.214) | 50 (15 - 100) | 36 | 47.986 (20.941) | 48.75 (12.5 - 85) | -2.188 (-9.974 : 5.599) | 0.59981 | -0.116 (-0.493 ; 0.262) | 14 |
| (QEEW) Need for recovery | 40 | 44.773 (31.248) | 40.909 (9.091 - 90.909) | 15 | 32.727 (24.003) | 27.273 (9.091 - 90.909) | -12.045 (-28.176 : 4.086) | 0.28375 | -0.408 (-1.02 ; 0.204) | 1 |
| (QEEW) Relationships with colleagues | 40 | 54.352 (11.603) | 55.556 (33.333 - 74.074) | 14 | 53.968 (8.309) | 51.852 (40.741 - 66.667) | -0.384 (-6.252 : 5.485) | 0.85002 | -0.035 (-0.658 ; 0.588) | 26 |
| (QEEW) Input/say/participaton | 40 | 61.042 (25.919) | 62.5 (12.5 - 100) | 13 | 53.526 (26.941) | 45.833 (12.5 - 91.667) | -7.516 (-25.307 : 10.275) | 0.35646 | -0.287 (-0.931 ; 0.356) | 4 |
| (LWC)Total score | 48 | 3 (2.518) | 3 (0 - 7) | 19 | 3.263 (2.579) | 3 (0 - 7) | 0.263 (-1.15 : 1.677) | 0.68812 | 0.104 (-0.438 ; 0.645) | 17 |

* p-values from Wilcoxon rank sum test with two sided continuity correction are not corrected for multiple comparisons and should be interpreted with caution.

# Supplemental Material - Retrospective T0 difference between individuals with a time between study inclusion and surgery of more or less than 6 months.

Table s5. Baseline (T0) characteristics for participants with more (>6m) and less (<6m) than 6 months between study inclusion and surgery

|  | Wait > 6 months group (N=102) | Wait < 6 months group (N=48) | Total with surgery date (N=150) |
| --- | --- | --- | --- |
| Age at inclusion |  |  |  |
| Mean (SD) | 64.4 (12.1) | 65.5 (12.3) | 64.8 (12.2) |
| Median [Min, Max] | 67.0 [18.0, 85.0] | 68.5 [32.0, 88.0] | 67.5 [18.0, 88.0] |
| Sex |  |  |  |
| Male | 52 (51.0%) | 32 (66.7%) | 84 (56.0%) |
| Female | 50 (49.0%) | 16 (33.3%) | 66 (44.0%) |
| Time between inclusion and surgery (months) |  |  |  |
| Mean (SD) | 11.2 (4.13) | 4.31 (1.17) | 8.98 (4.73) |
| Median [Min, Max] | 10.8 [6.10, 33.7] | 4.50 [1.00, 5.90] | 8.50 [1.00, 33.7] |
| Age of noticing hearing loss (years) |  |  |  |
| Mean (SD) | 38.9 (18.9) | 40.9 (16.8) | 39.6 (18.2) |
| Median [Min, Max] | 40.5 [0, 79.0] | 43.0 [4.00, 70.0] | 42.0 [0, 79.0] |
| Missing | 6 (5.9%) | 1 (2.1%) | 7 (4.7%) |
| Duration of hearing loss (years) |  |  |  |
| Mean (SD) | 25.9 (15.8) | 24.2 (13.3) | 25.3 (15.0) |
| Median [Min, Max] | 20.0 [2.00, 80.0] | 22.0 [2.00, 59.0] | 20.0 [2.00, 80.0] |
| Missing | 6 (5.9%) | 1 (2.1%) | 7 (4.7%) |
| Category etiology of hearing loss |  |  |  |
| Acquired | 16 (15.7%) | 9 (18.8%) | 25 (16.7%) |
| Not-syndromal | 22 (21.6%) | 7 (14.6%) | 29 (19.3%) |
| Syndromal | 6 (5.9%) | 0 (0%) | 6 (4.0%) |
| Unknown | 58 (56.9%) | 30 (62.5%) | 88 (58.7%) |
| Other | 0 (0%) | 2 (4.2%) | 2 (1.3%) |
| Paid or unpaid Work |  |  |  |
| Yes | 61 (59.8%) | 25 (52.1%) | 86 (57.3%) |
| No | 40 (39.2%) | 23 (47.9%) | 63 (42.0%) |
| Missing | 1 (1.0%) | 0 (0%) | 1 (0.7%) |
| Paid Work |  |  |  |
| Yes | 32 (31.4%) | 17 (35.4%) | 49 (32.7%) |
| No | 69 (67.6%) | 30 (62.5%) | 99 (66.0%) |
| Missing | 1 (1.0%) | 1 (2.1%) | 2 (1.3%) |
| Education |  |  |  |
| Higher Education | 32 (31.4%) | 26 (54.2%) | 58 (38.7%) |
| Secondary Education | 65 (63.7%) | 20 (41.7%) | 85 (56.7%) |
| Primary education | 4 (3.9%) | 2 (4.2%) | 6 (4.0%) |
| Missing | 1 (1.0%) | 0 (0%) | 1 (0.7%) |
| Living situation |  |  |  |
| Alone | 20 (19.6%) | 6 (12.5%) | 26 (17.3%) |
| with others (partner, children) | 81 (79.4%) | 42 (87.5%) | 123 (82.0%) |
| Missing | 1 (1.0%) | 0 (0%) | 1 (0.7%) |
| Unaided phonemescore(%) at 65 dB TBI |  |  |  |
| Mean (SD) | 3.27 (12.4) | 3.39 (10.6) | 3.31 (11.8) |
| Median [Min, Max] | 0 [0, 76.0] | 0 [0, 45.0] | 0 [0, 76.0] |
| Missing | 1 (1.0%) | 2 (4.2%) | 3 (2.0%) |
| Unaided phonemescore(%) at 65 dB non-TBI |  |  |  |
| Mean (SD) | 5.77 (14.5) | 5.00 (12.6) | 5.53 (13.9) |
| Median [Min, Max] | 0 [0, 79.0] | 0 [0, 51.0] | 0 [0, 79.0] |
| Missing | 5 (4.9%) | 3 (6.3%) | 8 (5.3%) |
| Unaided phonemescore(%) at 75 dB TBI |  |  |  |
| Mean (SD) | 6.92 (15.7) | 6.09 (15.2) | 6.66 (15.5) |
| Median [Min, Max] | 0 [0, 85.0] | 0 [0, 58.0] | 0 [0, 85.0] |
| Missing | 0 (0%) | 1 (2.1%) | 1 (0.7%) |
| Unaided phonemescore(%) at 75 dB non-TBI |  |  |  |
| Mean (SD) | 14.8 (21.5) | 10.4 (18.1) | 13.4 (20.6) |
| Median [Min, Max] | 0 [0, 88.0] | 0 [0, 60.0] | 0 [0, 88.0] |
| Missing | 3 (2.9%) | 2 (4.2%) | 5 (3.3%) |
| Unaided phonemescore(%) at 85 dB TBI |  |  |  |
| Mean (SD) | 12.0 (19.1) | 9.96 (19.3) | 11.3 (19.1) |
| Median [Min, Max] | 0 [0, 73.0] | 0 [0, 60.0] | 0 [0, 73.0] |
| Unaided phonemescore(%) at 85 dB non-TBI |  |  |  |
| Mean (SD) | 26.7 (26.2) | 19.7 (24.9) | 24.5 (25.9) |
| Median [Min, Max] | 24.0 [0, 84.0] | 0 [0, 69.0] | 18.0 [0, 84.0] |
| Hearing aid use |  |  |  |
| Yes | 101 (99.0%) | 46 (95.8%) | 147 (98.0%) |
| No | 1 (1.0%) | 2 (4.2%) | 3 (2.0%) |
| Aided phonemescore(%) at 55 dB TBI |  |  |  |
| Mean (SD) | 12.0 (17.1) | 8.82 (15.9) | 11.0 (16.7) |
| Median [Min, Max] | 0 [0, 73.0] | 0 [0, 66.0] | 0 [0, 73.0] |
| Missing | 7 (6.9%) | 4 (8.3%) | 11 (7.3%) |
| Aided phonemescore(%) at 55 dB non-TBI |  |  |  |
| Mean (SD) | 24.6 (21.9) | 18.7 (20.5) | 22.8 (21.6) |
| Median [Min, Max] | 21.0 [0, 76.0] | 12.0 [0, 63.0] | 19.0 [0, 76.0] |
| Missing | 12 (11.8%) | 7 (14.6%) | 19 (12.7%) |
| Aided phonemescore(%) at 65 dB TBI |  |  |  |
| Mean (SD) | 26.6 (24.3) | 23.4 (24.5) | 25.6 (24.3) |
| Median [Min, Max] | 30.0 [0, 87.0] | 16.5 [0, 82.0] | 24.5 [0, 87.0] |
| Aided phonemescore(%) at 65 dB non-TBI |  |  |  |
| Mean (SD) | 47.5 (26.1) | 41.8 (27.6) | 45.7 (26.6) |
| Median [Min, Max] | 53.3 [0, 91.0] | 46.0 [0, 85.0] | 52.0 [0, 91.0] |
| Aided phonemescore(%) at 75 dB TBI |  |  |  |
| Mean (SD) | 34.1 (27.4) | 28.8 (29.5) | 32.4 (28.1) |
| Median [Min, Max] | 42.0 [0, 96.0] | 30.0 [0, 91.5] | 36.0 [0, 96.0] |
| Aided phonemescore(%) at 75 dB non-TBI |  |  |  |
| Mean (SD) | 57.0 (27.4) | 49.5 (26.2) | 54.6 (27.2) |
| Median [Min, Max] | 63.0 [0, 97.0] | 56.5 [0, 88.0] | 61.8 [0, 97.0] |

Table s6. Baseline (T0) outcome scores for participants with valid scores that had more (>6months) and less (<6months) than 6 months between study inclusion and surgery

| Names | N (Wait > 6 months group) | Wait > 6 months group mean (SD) | Wait > 6 months group median (ranges) | N (Wait < 6 months group) | Wait < 6 months group mean (SD) | Wait < 6 months group median (ranges) | Difference (95% CI) | p-value* | Cohen's D (95% CI) | Cohens D rank |
| --- | --- | --- | --- | --- | --- | --- | --- | --- | --- | --- |
| (IPA) Autonomy indoors | 101 | 0.424 (0.519) | 0.143 (0 - 2) | 48 | 0.366 (0.636) | 0 (0 - 3) | -0.058 (-0.268 : 0.151) | 0.21531 | -0.104 (-0.451 ; 0.242) | 14.0 |
| (IPA) Autonomy outdoors | 101 | 1.531 (0.781) | 1.4 (0 - 3.2) | 48 | 1.271 (0.833) | 1.2 (0 - 4) | -0.26 (-0.544 : 0.025) | 0.03970 | -0.326 (-0.674 ; 0.023) | 5.0 |
| (IPA) Family role | 101 | 0.876 (0.769) | 1 (0 - 3) | 48 | 0.693 (0.855) | 0.429 (0 - 3.714) | -0.182 (-0.471 : 0.107) | 0.08411 | -0.228 (-0.576 ; 0.119) | 7.0 |
| (IPA) Social life and relationships | 101 | 1.593 (0.707) | 1.571 (0 - 3.429) | 48 | 1.357 (0.777) | 1.286 (0.143 - 3.857) | -0.236 (-0.499 : 0.028) | 0.02876 | -0.322 (-0.671 ; 0.026) | 6.0 |
| (IPA) Work and Education | 45 | 2.418 (0.918) | 2.333 (1 - 4.333) | 21 | 1.865 (1.105) | 2 (0.167 - 3.5) | -0.553 (-1.117 : 0.011) | 0.06668 | -0.564 (-1.101 ; -0.027) | 3.0 |
| (CPHI) Maladaptive Behavior | 101 | 3.438 (0.739) | 3.5 (2 - 5) | 48 | 3.383 (0.784) | 3.375 (1.75 - 5) | -0.055 (-0.324 : 0.213) | 0.67076 | -0.073 (-0.42 ; 0.273) | 20.0 |
| (CPHI) Verbal Strategies | 101 | 3.236 (0.796) | 3.25 (1.75 - 5) | 48 | 3.216 (0.821) | 3.25 (1.25 - 5) | -0.02 (-0.303 : 0.263) | 0.89321 | -0.025 (-0.372 ; 0.321) | 28.0 |
| (CPHI) Nonverbal Strategies | 101 | 3.874 (0.735) | 4 (1.429 - 5) | 48 | 3.815 (0.795) | 4 (1.571 - 5) | -0.059 (-0.329 : 0.212) | 0.72173 | -0.078 (-0.424 ; 0.269) | 18.5 |
| (CPHI) Self-Acceptance | 101 | 3.789 (0.89) | 4 (1.167 - 5) | 48 | 3.701 (0.908) | 3.833 (1.333 - 5) | -0.087 (-0.402 : 0.227) | 0.57412 | -0.098 (-0.444 ; 0.249) | 15.0 |
| (CPHI) Acceptance of Loss | 101 | 3.626 (0.732) | 3.75 (1.75 - 5) | 48 | 3.615 (0.776) | 3.625 (1.25 - 5) | -0.012 (-0.277 : 0.254) | 0.94970 | -0.016 (-0.362 ; 0.331) | 29.0 |
| (CPHI) Stress, and Withdrawal | 101 | 2.499 (0.756) | 2.533 (1.133 - 4.533) | 48 | 2.45 (0.779) | 2.433 (1 - 5) | -0.049 (-0.318 : 0.22) | 0.71298 | -0.064 (-0.411 ; 0.282) | 21.0 |
| (NCIQ) Basic sound perception | 100 | 26.361 (14.343) | 25 (0 - 72.5) | 47 | 29.125 (16.151) | 27.5 (0 - 67.5) | 2.764 (-2.723 : 8.252) | 0.32716 | 0.185 (-0.165 ; 0.535) | 10.0 |
| (NCIQ) Advanced sound perception | 100 | 31.768 (15.096) | 30 (0 - 72.5) | 47 | 32.533 (18.649) | 30 (0 - 90) | 0.764 (-5.433 : 6.962) | 0.93869 | 0.047 (-0.303 ; 0.396) | 24.0 |
| (NCIQ) Speech production | 98 | 70.855 (17.791) | 72.5 (12.5 - 100) | 46 | 73.913 (18.307) | 73.75 (20 - 100) | 3.058 (-3.388 : 9.505) | 0.29833 | 0.17 (-0.184 ; 0.524) | 11.0 |
| (NCIQ) Self esteem | 100 | 50.447 (18.316) | 51.25 (8.333 - 97.5) | 47 | 52.816 (18.51) | 50 (15 - 90) | 2.37 (-4.113 : 8.852) | 0.54404 | 0.129 (-0.221 ; 0.479) | 13.0 |
| (NCIQ) Activity limitations | 99 | 48.797 (17.894) | 47.222 (10 - 90) | 47 | 47.801 (20.089) | 47.5 (2.5 - 95) | -0.996 (-7.836 : 5.844) | 0.87844 | -0.053 (-0.404 ; 0.297) | 22.0 |
| (NCIQ) Social interaction | 100 | 48.892 (17.586) | 50 (12.5 - 92.5) | 47 | 50.558 (17.813) | 50 (7.143 - 92.5) | 1.666 (-4.568 : 7.9) | 0.47096 | 0.094 (-0.255 ; 0.444) | 16.0 |
| HUI3 Score | 101 | 0.453 (0.18) | 0.474 (-0.08 - 0.849) | 48 | 0.482 (0.23) | 0.515 (-0.228 - 0.825) | 0.029 (-0.046 : 0.104) | 0.21830 | 0.149 (-0.198 ; 0.496) | 12.0 |
| EQ5D VAS score | 99 | 79.828 (13.699) | 80 (40 - 100) | 47 | 78.745 (14.483) | 80 (30 - 100) | -1.084 (-6.096 : 3.929) | 0.75852 | -0.078 (-0.428 ; 0.273) | 18.5 |
| EQ5D Value | 99 | 0.843 (0.148) | 0.883 (0.34 - 1) | 47 | 0.836 (0.205) | 0.883 (0.012 - 1) | -0.007 (-0.074 : 0.06) | 0.84373 | -0.042 (-0.392 ; 0.308) | 25.0 |
| ICECAP-A Value | 100 | 0.848 (0.118) | 0.88 (0.369 - 1) | 47 | 0.858 (0.142) | 0.888 (0.364 - 1) | 0.01 (-0.037 : 0.058) | 0.18535 | 0.08 (-0.269 ; 0.43) | 17.0 |
| (HIISOP) Communication strategy | 78 | 19.103 (3.626) | 20 (5 - 25) | 37 | 19.865 (3.63) | 20 (12.5 - 25) | 0.762 (-0.682 : 2.207) | 0.15950 | 0.21 (-0.186 ; 0.607) | 8.0 |
| (HIISOP) Relationship and emotions | 78 | 22.981 (12.523) | 22.5 (0 - 55) | 37 | 22.568 (12.183) | 25 (0 - 55) | -0.413 (-5.305 : 4.478) | 0.94975 | -0.033 (-0.429 ; 0.362) | 27.0 |
| (HIISOP) Social impact | 78 | 7.885 (5.613) | 7.5 (0 - 20) | 37 | 8.176 (5.61) | 7.5 (0 - 20) | 0.291 (-1.942 : 2.525) | 0.78337 | 0.052 (-0.344 ; 0.447) | 23.0 |
| (HIISOP) Total | 78 | 49.968 (18.258) | 48.75 (15 - 95) | 37 | 50.608 (18.366) | 52.5 (15 - 100) | 0.64 (-6.657 : 7.937) | 0.75058 | 0.035 (-0.361 ; 0.431) | 26.0 |
| (QEEW) Need for recovery | 26 | 46.853 (31.354) | 45.455 (9.091 - 90.909) | 14 | 40.909 (31.843) | 27.273 (9.091 - 90.909) | -5.944 (-27.511 : 15.623) | 0.54626 | -0.189 (-0.861 ; 0.484) | 9.0 |
| (QEEW) Relationships with colleagues | 26 | 52.849 (10.763) | 53.704 (33.333 - 70.37) | 14 | 57.143 (12.97) | 61.111 (37.037 - 74.074) | 4.294 (-4.106 : 12.694) | 0.23486 | 0.371 (-0.305 ; 1.048) | 4.0 |
| (QEEW) Input/say/participaton | 26 | 54.968 (23.864) | 54.167 (12.5 - 95.833) | 14 | 72.321 (26.635) | 77.083 (29.167 - 100) | 17.353 (-0.217 : 34.924) | 0.04212 | 0.698 (0.009 ; 1.388) | 2.0 |
| (LWC)Total score | 31 | 2.387 (2.39) | 1 (0 - 7) | 17 | 4.118 (2.421) | 5 (0 - 7) | 1.731 (0.25 : 3.211) | 0.02610 | 0.721 (0.096 ; 1.346) | 1.0 |

* p-values from Wilcoxon rank sum test with two sided continuity correction are not corrected for multiple comparisons and should be interpreted with caution.
